# Non-Celiac Gluten/Wheat Sensitivity: Clinical Characteristics and Microbiota and Mycobiota Composition by Response to the Gluten Challenge Test

**DOI:** 10.3390/nu13041260

**Published:** 2021-04-12

**Authors:** Valentina Ponzo, Ilario Ferrocino, Ilaria Goitre, Marianna Pellegrini, Mauro Bruno, Marco Astegiano, Gianni Cadario, Eleonora Castellana, Fabio Bioletto, Maria Rita Corvaglia, Patrizia Malfa, Luca Cocolin, Ezio Ghigo, Simona Bo

**Affiliations:** 1 Department of Medical Sciences, University of Torino, 10126 Torino, Italy; valentina.ponzo@unito.it (V.P.); ilaria.goitre@libero.it (I.G.); mariannapellegrini87@gmail.com (M.P.); fabio.bioletto@unito.it (F.B.); ezio.ghigo@unito.it (E.G.); 2 Department of Agricultural, Forestry and Food Science, University of Torino, 10095 Torino, Italy; mariarita.corvaglia@unito.it (M.R.C.); lucasimone.cocolin@unito.it (L.C.); 3 Gastroenterology and Digestive Endoscopy Unit, “Città della Salute e della Scienza” Hospital, 10126 Torino, Italy; mbruno@cittadellasalute.to.it (M.B.); mastegiano@cittadellasalute.to.it (M.A.); 4 Allergology and Clinical Immunology Unit, “Città della Salute e della Scienza” Hospital, 10126 Torino, Italy; gcadario@cittadellasalute.to.it; 5 Hospital Pharmacy, “Città della Salute e della Scienza” Hospital, 10126 Torino, Italy; ecastellna@cittadellasalute.to.it; 6 Proge Farm, 28100 Novara, Italy; patrizia.malfa@gmail.com

**Keywords:** gluten sensitivity, mental status, gut microbiota, gut mycobiota

## Abstract

The aims of this observational “proof-of-concept” study were to analyze the clinical/psychological characteristics and gut microbiota/mycobiota composition of individuals with suspected non-celiac gluten/wheat sensitivity (NCGS/WS) according to responses to the double-blind-placebo-controlled (DBPC) crossover gluten challenge test. Fifty individuals with suspected NCGS/WS were subjected to the DBPC challenge test; anthropometric measurements, psychometric questionnaires, and fecal samples were collected. Twenty-seven (54%) participants were gluten responsive (NCGS), and 23 were placebo responsive, with an order effect. NCGS individuals displayed a significantly lower risk of eating disorders and a higher mental health score when compared to placebo-responsive participants, confirmed by multiple logistic regression analyses (OR = 0.87; 95% CI 0.76–0.98, *p* = 0.021, and OR = 1.30; 95% CI 1.06–1.59, *p* = 0.009, respectively). Principal coordinate analyses based on microbiota composition showed a separation by the DBPC response (*p* = 0.039). For *Bacteroides* (*p* = 0.05) and *Parabacteroides* (*p* = 0.007), the frequency of amplicon sequence variants was lower, and that for *Blautia* (*p* = 0.009) and *Streptococcus* (*p* = 0.004) was higher in NCGS individuals at multiple regression analyses. No difference in the mycobiota composition was detected between the groups. In conclusion, almost half of the individuals with suspected gluten sensitivity reported symptoms with placebo; they showed lower mental health scores, increased risk for eating disorders, and a different gut microbiota composition.

## 1. Introduction

Non-celiac gluten/wheat sensitivity (NCGS/WS) is a clinical entity characterized by intestinal and extraintestinal symptoms induced by gluten ingestion in the absence of wheat allergy (WA) or celiac disease (CD) [1,2]. Components of wheat other than gluten have also been suggested to contribute to NCGS/WS symptoms. Specifically, there is supportive data from clinical trials for the potential involvement of fermentable oligo-, di-, monosaccharides, and polyols (FODMAPs) [1,2]. Several different substances other than gluten, such as fermentable oligo-, di-, monosaccharides, and polyols (FODMAPs), amylase trypsin inhibitors (ATIs), wheat germ agglutinin (WGA), and glyphosate may be involved in its pathogenesis [1,2]. Gluten peptides are resistant to gastrointestinal proteolytic degradation and may elicit immunogenic responses, alter the intestinal permeability, and promote oxidative stress and pro-inflammatory and cytotoxic effects [1,2]. Indeed, FODMAPs may act by osmotic activity, fluid retention, fermentation and gas production, and ATIs/WGA through immune responses, pro-inflammatory effects, and intestinal barrier disruption, respectively [1,2].

The pathogenesis of NCGS/WS is still uncertain; a higher intestinal expression of toll-like receptors suggesting a stronger implication of the innate immune mechanisms, the infiltration of immune cells in the gut mucosa, increased expression of antibodies, and dysregulated gene expression profiling and epigenetic mechanisms have been described [1,2,3,4,5,6,7,8,9]. Furthermore, impaired intestinal barrier and permeability and gut dysbiosis have been advocated as key factors by several studies [1,2,4,10,11]. In particular, the microbiota composition of NCGS/WS individuals significantly differed from that of healthy individuals [12,13]. Indeed, the causal association between dysbiosis and NCGS/WS is still unclear due to the controversial results, the high interindividual microbiota variability, and the different methodologies employed in the gut microbiota analysis [1], and there are still no available data about the mycobiota composition. The nocebo effect of wheat/gluten ingestion might also play a role, above all in self-reported diagnoses, due to the lack of specific diagnostic criteria and the widespread negative pressure on social networks about the toxic effect of gluten [14]. However, only few studies evaluated the psychological characteristics of the participants [15,16]. Furthermore, the double-blind placebo-controlled (DBPC) crossover gluten challenge test, which remains at present the gold standard test for NCGS/WS diagnosis, is cumbersome and not easy to perform in daily clinical practice. Indeed, many studies referred to self-reported diagnosis [3,9,10,13,17], but only 8% to 34% of the individuals with self-reported gluten sensitivity worsened their symptoms after gluten ingestion, thus suggesting that self-reporting overemphasized the syndrome [18,19,20,21,22].

The aim of the present observational “proof-of-concept” study was to analyze the clinical and psychological characteristics and the gut microbiota and mycobiota of individuals seeking medical help for suspected NCGS/WS according to their response to the DBPC gluten challenge test.

## 2. Materials and Methods

### 2.1. Participants

**Figure 1:**
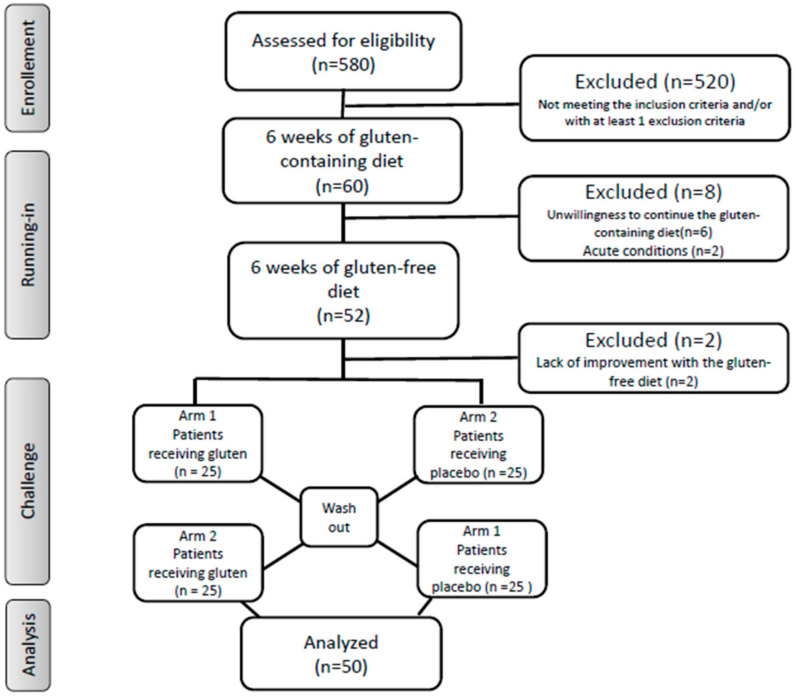
Flow of the study.

The individuals seeking medical assistance for any form of gluten/wheat intolerance at the outpatient gastroenterology and/or allergology clinics of the “Città della Salute e della Scienza” Hospital of Torino, were evaluated for enrollment, starting from January 2018 (Figure 1).

Inclusion criteria included the following: adults (age ≥ 18 years), and gluten/wheat sensitivity demonstrated by the following self-reported conditions (i) intestinal and extraintestinal symptoms associated within hours or days after the ingestion of gluten-containing food, (ii) a clear benefit while they were on a gluten-free diet, (iii) relapse of symptoms with the ingestion of gluten-containing foods.

Exclusion criteria were as follows: presence of CD, WA, (irritable bowel syndrome, IBS), and other gastrointestinal diseases; *H. pylori* infection; lactose intolerance; history of gastrointestinal surgery; liver/pancreatic diseases; active or recent infectious diseases; known psychiatric diseases; any systemic diseases; pregnancy or breastfeeding; active use of antibiotics, probiotics, immunosuppressive drugs, non-steroidal anti-inflammatory drugs, or corticosteroids; CD in first-degree relatives; inability to give written informed consent.

CD was diagnosed in the presence of CD compatible clinical symptoms, positive IgA tissue transglutaminase antibodies and/or endomysial antibodies, and villous atrophy of the duodenal mucosa according to the Marsh–Oberhuber criteria [23]. WA was diagnosed in the presence of positive IgE-mediated immune-allergy tests to wheat (skin prick tests and/or specific serum IgE detection). IBS was diagnosed according to the Roma IV clinical criteria [24,25].

The first consecutive 60 individuals with possible NCGS/NCWS and without CD, WA, IBS, and the other exclusion criteria were enrolled for the present study.

### 2.2. Ethical Issues

All the participants gave their written informed consent to participate. The local Ethics Committee for Human Research approved the study protocol.

### 2.3. Outcomes

The primary outcome was evaluating the participants’ response to gluten by assessing their symptoms as measured by the modified version of the Gastrointestinal Symptom Rating Scale (GSRS), according to the Salerno Experts Criteria [26] after the DBPC crossover gluten challenge.

Secondary outcomes were evaluating the clinical, psychological characteristics, and the gut microbiota and mycobiota composition of participants by their responses to the gluten challenge.

### 2.4. First Step: Dietary Changes

Two registered, trained dieticians evaluated all patients at baseline, gave dietary recommendations, and checked diet compliance during the whole test. Participants were encouraged to immediately contact the dieticians by phone in case of any doubt or problem related to the diet.

During the nutritional evaluation, anthropometrical parameters, nutritional status, and usual dietary patterns were evaluated. The same trained dieticians assessed the usual dietary intake before the experiment and checked every 2 weeks the adherence to the given dietary recommendations during the whole experiment (both during the gluten-containing diet and during the gluten-free, low-FODMAPs diet) by collecting an accurate food history for each participant by a standardized interview.

Weight was measured using a mechanical column scale (SECA model 711, Hamburg, Germany) with the participants wearing light clothes and no shoes to the nearest 0.1 kg, height was measured to the nearest 0.1 cm with a stadiometer (SECA 220 measuring rod, Hamburg, Germany), and waist circumference was assessed using a plastic tape meter at the umbilicus level.

Arterial blood pressure values were measured on the left arm, in a sitting position, after at least 10 min of rest, with a mercury sphygmomanometer with appropriate cuff sizes (ERKA Perfect-Aneroid, Germany). Two measurements were taken with the arm supported at heart level, and the values reported were the means of the two.

The first step included a period with a gluten-containing diet (at least 10 g gluten/day, corresponding to around four slices of bread) for six weeks (Figure 1). The self-administered modified version of the GSRS was compiled by all participants during the last week of this period [26]. It was a 24-item questionnaire including both gastrointestinal (abdominal discomfort or pain, bloating, diarrhea, constipation, etc.) and extra-intestinal (headache, numbness in the limbs, fatigue, musculoskeletal pain, foggy mind, dermatitis, etc.) symptoms. The participants identified one to three main symptoms that were quantitatively assessed using a Numerical Rating Scale (NRS) with a score ranging from 1 (mild) to 10 (severe), according to the Salerno Experts Criteria [26]. The dieticians calculated the symptom scores.

Then, a six-week, gluten-free, low-FODMAPs diet was administered to all participants by the same dieticians, with detailed verbal and written explanations. During a 1 h session, the gluten-free diet was illustrated by the dietitians who provided oral and written recommendations, a list of allowed and unallowed foods and instructions on how to read the food labels. Patients were requested to avoid foods with a high content of FODMAPs, and a comprehensive list of foods with high and low FODMAPs content was supplied including fruits, vegetables, cereals, beverages, and condiments to be limited or consumed, respectively [27].

The modified GSRS questionnaire was compiled during the last week of this second period. A decrease of at least 30% of the baseline score was considered a positive response. Diet adherence during the different phases of the study was checked by phone contact by the dieticians every 2 weeks. Adherence to the gluten-containing diet and to the gluten-free, low-FODMAPs diet, respectively, was considered adequate if at least 10 g/day gluten was consumed, and <10 mg/day gluten and <3 g/day FODMAPs were consumed based on the participant-reported diet [28].

Out of the 60 enrolled participants, there were six dropouts because of unwillingness to continue the gluten-containing diet, two with acute conditions occurring during the first step and requiring drugs, and two who failed to show a symptomatic improvement with the gluten-free diet (Figure 1). Therefore, 50 individuals remained for the second step of the study, i.e., the DBPC crossover gluten challenge.

### 2.5. Second Step: (DBPC) Crossover Gluten Challenge Test

During the following step, the participants continued the same diet (gluten-free, low-FODMAPs diet) and the DBPC challenge test was performed: a one week challenge (either with gluten or placebo; 25 individuals with gluten first, 25 with placebo first), followed by a one week washout, and then a further one week crossover challenge (either with placebo or gluten, depending on randomization). Participants filled the above-described, modified GSRS questionnaire daily during the three weeks of the challenge. A variation of symptoms of at least 30% between gluten and placebo challenge discriminated a positive from a negative result [26].

A pharmacist, with no clinical involvement in the study, prepared the randomization sequence, according to a computer-generated series. All participants and study team members were blinded throughout the study. The participants were given the capsules (gluten or placebo) in identical anonymous bottles, marked with the number corresponding to the order of consumption (1 = first week; 3 = third week). Participants consumed for each of the two challenge weeks 10 capsules/day, ingested on no more than two occasions over the day, with a total daily amount of 5.6 g gluten, corresponding to the gluten content of 80 g pasta, in line with previous published protocols [22,29]. The gluten used was commercially available, carbohydrate-depleted wheat gluten (Bongiovanni s.r.l., Villanova Mondovì, Cuneo, Italy) and contained 75% protein, 0% crude fiber, 5% lipid, 15% starch, and 0% salt.

Placebo capsules contained rice starch for a total daily amount of 5.6 g; it has been used by most protocols because of its low fermentable capacity due to its rapid absorption [20,21,29]. Gluten and placebo preparations were undistinguishable in look, texture, and taste. The capsule consumption was checked by phone contact by the dieticians every week.

### 2.6. Questionnaires

Participants were administered the following questionnaires:

– The Minnesota Leisure Time Physical Activity Questionnaire: the physical activity level was calculated as the product of the duration and frequency of each activity (in h/week), weighted by an estimate of the metabolic equivalent (MET) of the activity and summed for the activities performed [30].
– The Hamilton rating scale for depression: mild depression was defined by scores ranging 8–17, moderate depression with scores ranging 18–24, and serious depression with scores >25 [31].
– The Hamilton rating scale for anxiety: mild anxiety was defined by a score <17, mild-to-moderate by scores ranging 18–24, and moderate-to-severe by scores >25 [32].
– ORTO-15 questionnaire: a 15 multiple-choice item tool investigating the obsessive attitude of the subjects in choosing, buying, preparing, and consuming food they consider to be healthy. A cutoff of 40 discriminates the presence or the absence of orthorexia [33].
– Eating Attitude test (EAT) 26: an abbreviated 26-item version of the EAT-40 relate to attitudes, beliefs, and behaviors concerning food, weight, and body shape. A cutoff score of 20 or above denotes the presence of disturbed eating behavior [34].
– Short-Form (SF)-12 health survey: to measure general health-related quality of life with different domains with scores ranging from 0 to 100 (higher values indicate a better quality of life) [35].

### 2.7. Fecal Samples

Fecal samples were self-collected at the end of the first step, before the DBPC challenge test, by the participants, who were specifically instructed. All materials were provided in a convenient, refrigerated, specimen collection kit. Patients were provided with sterile containers to collect the feces (VWR, Milan, Italy). The fecal samples were self-collected at home and immediately transferred to the sterile sampling containers using a polypropylene spoon (3 spoons of about 10 g) and stored at 4 °C. The specimens were transported to the laboratory within 12 hours of collection at a refrigerated temperature. Containers were immediately stored at −80 °C for DNA extraction. No storage medium was used.

### 2.8. Fecal DNA Extraction and Amplicon Target Sequencing

Total DNAs from fecal samples were extracted using the RNeasy Power Microbiome KIT (Qiagen, Milan, Italy) following the manufacturer’s instructions. One microliter of RNase (Illumina Inc., San Diego, CA, USA) was added to digest RNA in the DNA samples, with an incubation of 1 h at 37 °C. DNA was quantified using the QUBIT dsDNA Assay kit (Life Technologies, Milan, Italy) and standardized at 5 ng/μL. Two microliters of each DNA was amplified for microbiota analysis by using primers and conditions for the amplification of the V3–V4 region of the 16S rRNA gene [36]. The mycobiota was studied by the amplification of the D1–D2 domain of the 26S according to Mota-Gutierrez [37]. Pair-end sequencing (2 × 250 bp) was performed with a MiSeq Illumina instrument (Illumina Inc., San Diego, CA, USA) with V2 chemistry according to the manufacturer’s instructions.

### 2.9. Bioinformatics Analysis

After sequencing, raw reads were analyzed by using the Quantitative Insights into Microbial Ecology (QIIME) 2 [38]. Primers and adapters were first trimmed by using Cutadapter and then quality filtered using the DADA2 algorithm [39], removing low-quality bases, chimeric sequences, and sequences shorter than 300 bp by using the DADA2 denoise-paired plug in of QIIME2. Amplicon sequence variants (ASVs) generated by DADA2 were used for taxonomic assignment using the QIIME feature-classifier plugin against the Greengenes 16S rRNA gene database for the microbiota and the manually build database for the mycobiota [37]. Taxonomy assignment for 16S and 26S was double checked on BLAST suite tools.

### 2.10. Accession Numbers

The data generated by sequencing were deposited in the National Center for Biotechnology Information (NCBI) Sequence Read Archive (SRA) and are available under the BioProject Accession Number PRJNA701870.

### 2.11. Statistical Analyses

Normally distributed variables were presented as mean ± standard deviation, while non-normally distributed variables were presented as median (range interquartile). The Kolmogorov–Smirnov test was used for the assessment of normality. Variables were compared by the t-Student test, chi-square test, or Mann–Whitney U test, as appropriate.

QIIME2 diversity script was used to perform alpha and beta diversity analysis. A weighted UniFrac distance matrix was generated by QIIME2 and used both to build the principal coordinate analysis (PCoA) and to perform the permutational multivariate analysis of similarities (ANOSIM) by the “vegan” package in R environment. Box plots represented the interquartile range between the first and the third quartile, with the error bars showing the lowest and the highest value. The random forest model was performed by QIIME2 using the q2-sample-classifier plugin in order to identify variable importance by using the microbiota and mycobiota ASV tables. The receiver operating characteristic (ROC) curve and accuracy value (AUC) were calculated in order to verify the classification accuracy of a machine-learning model.

Multiple logistic regression analyses were performed to evaluate the associations between the responses to the gluten challenge test (dependent variable) and the variables significantly different at univariate analyses, adjusted for age, gender, and education level. In case specific ASVs were not found in the gut microbiota of one of the two groups, standard errors and p-values were computed by a resampling method (the jackknife technique) (STATA 16, StataCorp, College Station, TX, USA).

## 3. Results

The adherence to the given dietary recommendations (either to the gluten-containing diet or to the gluten-free, low-FODMAPs diet) was considered adequate for all the 50 enrolled participants. All the subjects took all the capsules, too. Median values of scores from the modified Gastrointestinal Symptom Rating Scale (GSRS) questionnaire were 12.0 during the gluten-containing diet and 4.0 during the gluten-free, low-FODMAPs diet.

### 3.1. Characteristics of the Participants

Most of participants were females, around 40 years old, with normal body mass index (BMI) and a high level of education (Table 1). All displayed anxiety, which was mostly moderate to severe. Depression and orthorexia criteria were achieved in around 40% of the participants, while only a minority showed an increasing risk for eating disorders. The reported health status, in particular the mental status, was low (Table 1).

### 3.2. Response to the DBPC Gluten Challenge Test

Twenty-seven out of fifty individuals (54%) had a symptomatic relapse during the gluten ingestion and were considered as having NCGS. Twenty-three out of fifty participants (46%) had a negative response and reported worsening of their symptoms after consuming the placebo. The median (interquartile range) of the scores of the reported symptoms after gluten and placebo in the two groups are reported in Table 2.

The most frequent gastrointestinal symptoms in NCGS were abdominal pain, bloating, and meteorism, while in individuals responsive to placebo they were bloating, abdominal distension, and meteorism. The extra-gastrointestinal symptoms were lesions of the oral cavity, dermatitis, and headache in gluten-responsive individuals, and headache, dermatitis, and pain in the limbs in placebo-responsive individuals (Table 2).

Data suggested the presence of an order effect. In fact, overall, 32/50 patients (64%) reported worse symptoms during the first week compared to the third week (*p* = 0.048 with respect to the null hypothesis of 50%). Being exposed to gluten rather than placebo during the first week of the test was a predictive factor for receiving a final diagnosis of NCGS; in fact, among patients who were first assigned to gluten consumption, 17/25 (68%) received a diagnosis of NCGS, while among patients who were first assigned to placebo consumption, only 10/25 (40%) received a diagnosis of NCGS.

No significant difference was evident between the two groups for age, gender, anthropometric characteristics, level of education, and exercise (Table 1). Individuals with a negative response to the DBPC gluten challenge showed increased, though, not significantly different, scores for depression, anxiety, and orthorexia. On the other hand, these subjects displayed a significantly higher risk of eating disorders and a lower mental health score when compared to individuals with NCGS (Table 1). In a multiple regression model, the association between positive response to the gluten test and reduced risk of eating disorders, and better mental health remained statistically significant (Table 3).

### 3.3. Analysis of the Microbiota

**Figure 2:**
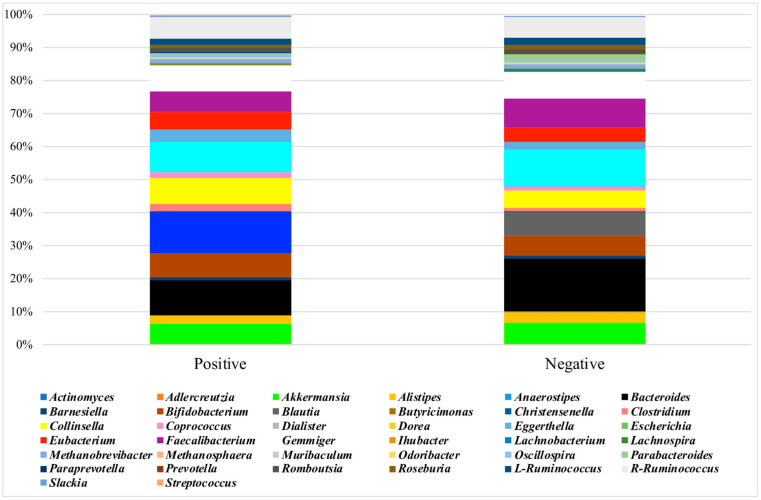
Mean frequency of ASV microbiota composition by response to the DBPC gluten challenge test.

**Figure 3:**
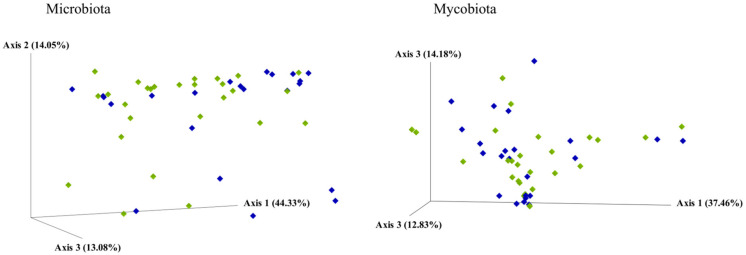
Principal coordinate analysis (PCoA) based on a weight UniFrac distance matrix by response to the DBPC gluten challenge test. Participants with a positive response (NCGS) are represented by green diamonds; participants with a negative response (placebo responsive) are represented by blue diamonds.

**Figure 4:**
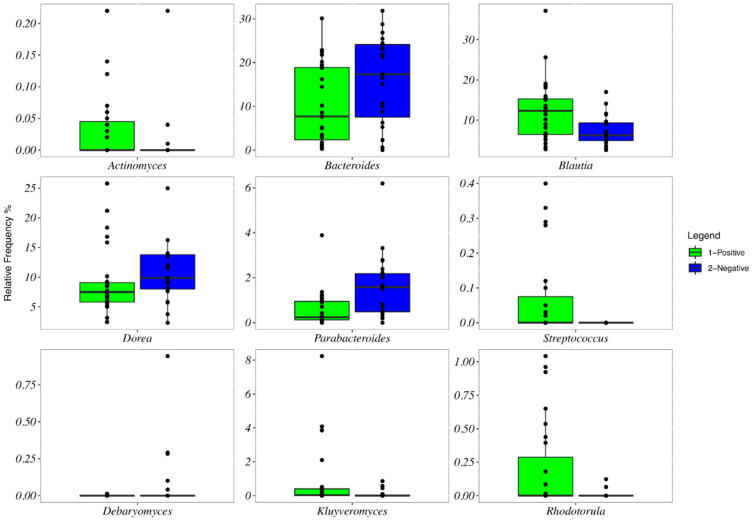
Box plots showing the relative frequencies of the genera of bacteria and fungi significantly different by the response to the DBPC gluten challenge. Participants with a positive response (NCGS) are represented by green bars; participants with a negative response (placebo responsive) are represented by blue bars. Boxes represent the interquartile range (IQR) between the first and third quartiles, and the line inside represents the median (second quartile). Whiskers denote the lowest and the highest values within 1.56 interquartile range from the first and third quartiles, respectively. Circles represent outliers beyond the whiskers.

The baseline microbiota α-diversity values were not significantly different between the two groups by the response to the gluten challenge test (Figure S1). The core microbiota was constituted in NCGS and placebo-responsive individuals, respectively, by *Blautia* (12% of the mean relative frequency and 7%), *Bacteroides* (10% and 16%), *Dorea* (9% and 11%), *Collinsella* (8% and 5%), *Gemmiger* (7% and 8%), *Bifidobacterium* (7% and 5%), R-*Ruminococcus* (7% and 6%), *Faecalibacterium* (6% and 9%), *Akkermansia* (6% and 7%), *Eubacterium* (5% and 4%), *Eggerthella* (4% and 2%), *Alistipes* (2% and 3%), *Clostridium* (2% and 1%), and L-*Ruminococcus*, *Coprococcus*, *Romboutsia*, and *Methanobrevibacter* (>1% in both groups) (Figure 2). *Dialister* and *Streptococcus* were absent in placebo-responsive subjects. By plotting the principal coordinate analysis (PCoA, Figure 3, left) of the weighed UniFrac distance matrix, a certain degree of separation between positive and negative subjects was evident, which was confirmed by the analysis of similarities (ANOSIM, *p* = 0.039). A box plot at genus level showed a significant reduction in the frequency of *Bacteroides*, *Dorea*, and *Parabacteroides* and a significant increase of *Blautia* and *Streptococcus* in individuals with NCGS (Figure 4). By using the random forest models to assess the predictive ability of the amplicon sequence variants (ASVs), we built the heatmap reporting the association between the gut bacteria genera and the response to the gluten challenge test. *Blautia* was the predominant ASV accounting for differences in the gut microbiome between participants with positive and negative responses (AUC = 0.92). In a multiple logistic regression analysis, the associations between the response to the gluten challenge and the relative frequency of *Blautia*, *Parabacteroides*, and *Streptococcus* remained statistically significant (Table 3).

### 3.4. Analysis of the Mycobiota

**Figure 5:**
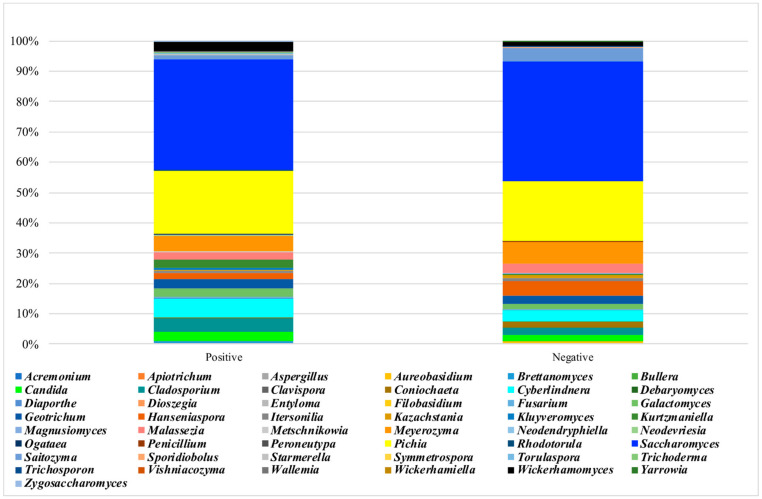
Mean frequency of ASV mycobiota composition by response to the DBPC gluten challenge test.

The mycobiota α-diversity values did not significantly differ by the response to the gluten challenge test (Figure S1). The core mycobiota was composed in NCGS and placebo-responsive individuals, respectively, by *Saccharomyces* (36% of the relative frequency and 38%), *Pichia* (20% and 19%), *Cyberlindnera* (6% and 3%), *Meyerozyma* (5% and 7%), *Cladosporium* (4% and 2%), *Wickerhamomyces* (3% and 1%), *Geotrichum* (3% and 2%), *Candida* (3% and 2%), *Kurtzmaniella* (2% and 0.2%), *Galactomyces* (2% and 1%), *Malassezia* (2% and 3%), *Hanseniaspora* (2% and 5%), *Saitozyma* (1% and 5%) and *Kluyveromyces* (1% and 0.05%) (Figure 5). No separation between subjects with positive and negative responses to the gluten challenge was observed by the weighed UniFrac distance matrix (Figure 3, left). Participants with NCGS showed a significant increase in the frequency of *Kluyveromyces* and *Rhodotorula* and a reduction of *Debaryomyces* (Figure 4). *Kluyveromyces* was the mycobiota ASV providing the highest discriminatory power between NCGS and placebo-responsive participants; however, in a multiple logistic regression analysis, no association with the response to the gluten challenge was observed (Table 3).

## 4. Discussion

Around half of the participants with a suspected intolerance to gluten/wheat reported worsening of their symptoms during the placebo consumption; those individuals showed an increased risk for eating disorders and a low mental health. Furthermore, an order effect was found, prompting a possible nocebo effect. Otherwise, the possibility that these subjects were intolerant to some other components of wheat and/or have a different form of hypersensitivity cannot be excluded.

### 4.1. Gluten Response

About half of our participants were gluten responsive, in line with previous studies reporting around one-third of positive responses to the DBPC gluten challenge [18,19,20,21,22]. The various prevalences of gluten challenge responsivity may be due to the high heterogeneity in the employed methodology across the studies, including dose and vehicle of gluten, duration of wash-out and gluten administration, the employed placebo, the different dietary recommendations given to participants, and the characteristics of the enrolled participants, since other studies included patients with irritable bowel syndrome (IBS) or dyspepsia [18,29,40,41,42]. The gluten and placebo we used were in line with previous published protocols [20,21,22,29]; the employed gluten amount was considered as being able to induce symptoms in hypersensitive patients [20]; rice starch has low fermentable capacity due to its ready absorbability and has been frequently used as a placebo [20,21,29]; the administration of a low-FODMAPs diet in the first step of the experiment to all participants likely allowed us to exclude IBS patients who usually respond to different exclusion diets [18]. Nevertheless, the DBPC challenge is far from perfect, even if it remains the gold standard for the NCGS diagnosis [21].

Our participants showed high scores for anxiety, depression, and orthorexia—the unhealthy obsession with healthy eating—in line with other studies [13,15,17,20]. Furthermore, their perceived quality of life was lower than estimates from the general Italian population [43], in accordance with literature [15,44]. Higher, though not statistically significant, levels of somatization were described in NCGS individuals with respect to healthy controls [16]. Gluten-associated neurological and psychiatric manifestations are well known, including depression, apathy, excessive anxiety, eating disorders, attention-deficit/hyperactivity disorder, and sleep complaints [1,2], and short-term exposure to gluten induced a feeling of depression, while a long-term gluten-free diet may reduce and normalize the severity of depressive symptoms [45,46]. To explain the neurocognitive symptoms of NCGS/WS individuals, the following mechanisms have been proposed: a gut–brain interaction (the brain–gut axis) with the existence of an increased intestinal permeability, the “leaky gut syndrome”, that allows gluten/wheat peptides to cross the gut barrier, and enter the blood–brain barrier, causing neuro-inflammation; the possible role of gut dysbiosis (the microbiota–brain–gut axis); the implication of gluten “exorphins”, opioid peptides derived from gluten digestion that can interfere with pain-inhibitory systems, emotionality, and memory processes; the binding of lipopolysaccharide (LPS) directly to toll-like receptor 4 (TLR4) on the luminal surface of brain blood vessels, resulting in local cytokine secretion in the brain and activation of the microglia [1,2,10,26,45]. Otherwise, it is possible that the greater anxiety, depression, and/or excessive care about diet might have induced an enhanced attention toward the symptom burden related to the ingestion of gluten-containing food, thus suggesting, at least in part of the participants, a nocebo effect. Accordingly, an order effect was found, particularly in individuals with a higher education level, suggesting strong anticipatory responses in the most educated participants. This effect has already been reported in other studies [18,20,22] and is considered the consequence of the psychological impact of entering into a clinical study and the strong preconception of food intolerances, with highest expectancy in the first period [22].

### 4.2. Gluten-Responsive Individuals

In patients with NCGS/WS, a systemic immune activation has been demonstrated; it was documented by increased serum levels of soluble CD14 and LPS-binding protein, as well as antibody reactivity to bacterial LPS and flagellin [10], enhanced expression of Toll-like receptor 2 (TLR2) [5], increased antibodies against gluten proteins [17,47], raised interferon gamma, interleukin (IL)-1 beta, IL-2, IL-12, IL-15, transforming growth factor (TNF) beta1, and TNF-α-expressing duodenal cells [3,29], increased serum levels of IL-8 and IL-15 [48], moderate intra-epithelial infiltration of eosinophils and lymphocytes, with increased inflammatory and regulatory CD4+ cells [6,13,17,47,49,50], and a high frequency of associated autoimmune diseases [7,51]. It has been hypothesized that individuals with NCGS/WS with a prevalent pathogenetic role for the FODMAPs, instead of gluten, may be a different patient population with less-prominent immunologic characteristics [6]. Furthermore, intestinal epithelial damage has been reported owing to the elevated circulating levels of fatty-acid-binding protein 2 (FABP2), a protein specific to intestinal epithelial cells, in correlation with the immune responses to microbial products [10], and the upregulation of claudin-4 in the intestinal mucosa [5]. Data relative to intestinal permeability are, however, conflicting, since either a lower [5] or a higher permeability with microbial product translocation has been reported [10,52]. 

Only a few studies have examined the gut microbiota of patients with NCGS/WS [12]. Increased relative abundance of the genus *Actinobacillus* (Gammaproteobacteria) and *Finegoldia* (Clostridia) and members of the Ruminococcaceae family were found in the duodenum and feces of NCGS/WS patients, without differences in α-diversity [12]. Furthermore, an increase in the duodenal *Pseudomonas* after four weeks of gluten-free diet was reported in these patients, and the authors hypothesized that some members of *Pseudomonas* can act as protective agents with low abundance prompting a higher sensitivity to dietary allergens [11]. Higher relative abundance of Firmicutes, in particular, the microbial families of Ruminococcaceae and Peptostreptococcaceae and reduced Porphyromonadaceae (Bacteroidetes) were described in NCGS individuals when compared to controls; the gluten-free diet significantly diminished Firmicutes and enriched the family of Bacteroidaceae (Bacteroidetes) in NCGS individuals only, and the low-FODMAPs diet induced a decrease in Bifidobacteriaceae (Actinobacteria) [13]. Intriguingly, the microbiota in NCGS patients has been reported to be more susceptible to nutrient changes than in healthy controls [13]. Similarly, a lower abundance of the Actinobacteria phylum was found after the consumption for seven days of a transgenic low-gliadin wheat bread in NCGS patients, with an increase in the relative abundance of *Roseburia* and *Faecalibacterium* and a decrease in the *Bacteroides*, *Blautia*, *Dorea*, *Coprococcus*, and *Collinsella* genera, with an overall microbial profile with anti-inflammatory properties [53]. 

The evaluation of the microbiota of our participants was performed after 6 weeks of gluten-free, low-FODMAPs diet; therefore, we were not able to determine possible changes in its composition with respect to the gluten-containing diet, and our results are difficult to compare with other studies, where nutritional intakes have not been standardized. Nevertheless, a distinctive gut microbiota composition was evident between gluten-responsive and placebo-responsive individuals. In particular, the reduced relative abundance of *Bacteroides* and *Parabacteroides* was in line with the beneficial changes observed in NCGS individuals only after the gluten-free diet [53], and the higher relative abundance of streptococci was in accordance with findings in patients with functional dyspepsia [54]. Literature data are, however, controversial, since either bacteria from the *Bacteroides* genus have been shown to promote beneficial anti-inflammatory and probiotic activities [55,56,57] or an increase in the gut *Bacteroides* has been correlated with the development of pathological conditions, such as CD and IBD [58]. 

We observed a significantly higher relative abundance of *Blautia* in gluten-responsive participants, in line with what was reported in IBS patients [59,60], which was attributed to the capacity of *Blautia* of utilizing the large gas amount produced in the intestine of these patients [61]. Indeed, the potential role of the genus *Blautia*, one of the major butyrate-producing ones in the human gut, is still unclear in patients with bowel diseases [62]. 

The human mycobiota is a neglected component of the microbiota; most species derive from diet, and a low gut abundance characterizes healthy individuals, while some fungi contribute to the aggravation of the inflammatory response in gastrointestinal diseases; in particular, fungal dysbiosis resulted in enhanced immune responses [63,64,65]. The genera *Candida, Saccharomyces, Cladosporium, Penicillium, Pichia, Malassezia*, and *Aspergillus* are the most abundant fungi in the gut [66,67]. In our participants, we found a lower richness in *Saccharomyces*, *Candida*, and *Malassezia* than reported in literature [63,65,68]; this may be due to the gluten-free, low-FODMAPs diet of our participants, which was low in carbohydrates and fermented foods. The mycobiota has been characterized in various gastrointestinal diseases and conditions, such as inflammatory bowel diseases, gastrointestinal graft-versus-host diseases, colorectal cancer, and viral or alcoholic hepatitis [63,65,66,68,69,70], but no studies, to the best of our knowledge, had ever evaluated its composition in NCGS/WS. No significant difference in the mycobiota composition was observed between our gluten-responsive and placebo-responsive participants. The association between *Kluyveromyces* and NCGS was almost threefold higher, though not statistically significant. Accordingly, *Kluyveromyces* was found to be one of the few fungi identified in pediatric patients with inflammatory bowel diseases [71].

### 4.3. Placebo-Responsive Individuals

Around half of our participants reported symptoms during the consumption of the placebo. The differences in the reported symptoms between open (during the step 1 of the study) and blinded (during the DBPC challenge test) gluten consumption were strongly in support of a nocebo effect in our individuals. The nocebo effect has been reported in up to 40% of patients with suspected NCGS in a metanalysis of 10 double-blind, placebo-controlled, gluten challenge trials, comprising 1312 adults [21]. Among the possible causes, the authors suggested negative expectations, carryover effects in crossover trials, or the use of a placebo containing FODMAPs [21]. The perception of the benefits of gluten avoidance due to the potential harm of gluten is frequent among the general population, and up to 30% of individuals adopted gluten-free regimens without proper reasons [1,2]. In accordance with the possibility of fear of gluten toxic effect, our placebo-responsive participants showed a significant increased risk of eating disorders and a lower mental health status, and worse, though not statistically significant, scores for depression, anxiety, and orthorexia. 

Other possibilities should be considered. These individuals may be intolerant to other wheat components, such as amylase trypsin inhibitors (ATIs) or wheat germ agglutinin (WGA), as proposed by many authors [1,2]. ATIs are a family of proteins found in the endosperm of plant seeds, including rice, and they play a role in the natural defense against parasites and insects [72]. Orally ingested ATIs are resistant to proteases and heat and may trigger innate immune activation in intestinal cells by stimulating TLR4 [73]. Gluten-containing cereals showed higher bioactivity with a higher concentration of ATIs that activate TLR4 with respect to gluten-free cereals, such as rice, in which a low TLR4 stimulatory activity was found [74]. Indeed, available data are from in vitro animal studies without clear relevance or connection to NCGS/WS patients. The gluten challenge is an imperfect test, and the choice of an appropriate placebo is complex and often far from optimal. Rice starch was chosen as a placebo because it does not contain ATIs (which are instead present in the protein fraction of rice) and FODMAPs. Usually, rice starch is not associated with the induction of abdominal symptoms owing to its low fermentation capacity [20,21,29]. We cannot exclude the possibility that placebo-responsive individuals could be intolerant to rice starch because of the presence of sucrase–isomaltase deficiency (or sucrose intolerance), but the chance of having this condition is quite remote as it is a rare disease [75]. Finally, placebo-responsive individuals might have a reduced tolerance to the gelatin envelope of the capsules; however, since the envelopes were identical in the placebo and gluten capsules, the more-relevant adverse reactions with placebo remained unexplained.

The difference between the two groups suggested that gluten-sensitive and placebo-sensitive individuals were affected by two distinct conditions, with dysbiosis and a likely impaired immune response in the former, and a predominant nocebo effect in the latter. Recently, NCGS/WS has been considered as a multifactor-onset disorder, probably transient and preventable, related to quality and balance of the diet and not to the presence of gluten in itself [76]. 

Caution in the interpretation of these findings is required owing to the low sample size. This was a proof-of-concept study and, therefore, no explicit estimations of the sample size were made at the time of study design; however, in a post hoc analysis, given the observed results and considering 0.05 as significance level, this study had a power of 82% for the detection of differences in the mental health score. Most of our participants were women (86%), but this finding is in line with the gender distribution reported in literature. We have evaluated one fecal sample only, when participants were on a gluten-free low-FODMAPs diet, and the human microbiota tends to be resilient, i.e., returns to its original state, after being subjected to a perturbation. Indeed, the careful standardization of the dietary habits of the participants for a long enough period (12 weeks) reasonably eliminated the potential interferences on the gut microbiota due to dietary differences between groups. The lack of a control group did not allow us to define the presence of dysbiosis in our participants. However, imposing six weeks of a gluten-free, low-FODMAPs diet to healthy controls, in order to avoid confounders in the comparison of microbiota compositions, would be unacceptable and ethically incorrect. Further strengths of the present study were the careful selection of participants, in order to exclude other gastrointestinal diseases or confounding conditions; the double-blind-placebo controlled test, by using indistinguishable capsules; the good level of participant compliance to the given dietary recommendations and test challenge; and the novelty of the mycobiota analysis, which has never been studied before in these individuals.

## 5. Conclusions

In conclusion, around half of the individuals with suspected NCGS reported worsening of their symptoms during gluten consumption; they showed a reduced risk for eating disorders and a better mental health than placebo-responsive participants, prompting a possible nocebo effect in the latter. On the other hand, the gut microbiota composition of placebo-responsive individuals was different from that of gluten-responsive individuals. These results are worthy to be confirmed by further studies, testing in sequence different wheat components other than gluten.

## Figures and Tables

**Table 1:** Characteristics of the enrolled participants by their response to the double-blind placebo-controlled (DBPC) challenge.

	All ^c^	Positive ^c^ (NCGS)	Negative ^c^	P
Number	50	27	23	
Age (years)	42.2 ± 13.6	41.3 ± 14.8	43.3 ± 12.1	0.60
Male/Female	7/43	4/23	3/20	0.86
Smoking (actual or past) (%)	16.0	11.1	21.7	0.24
Education level				
Primary school (%)	16.0	19.2	13.0	
Secondary school (%)	36.0	26.9	43.5	
University degree (%)	48.0	53.9	43.5	0.31
Weight (kg)	64.0 ± 13.6	62.0 ± 9.7	66.3 ± 17.0	0.27
BMI (kg/m^2^) ^a^	23.7 ± 5.1	23.0 ± 3.2	24.6 ± 6.7	0.29
Waist circumference (cm)	85.5 ± 12.1	84.1 ± 8.6	87.0 ± 1.3	0.40
Systolic blood pressure (mmHg)	114.5 ± 13.4	115.4 ± 12.0	113.5 ± 15.2	0.61
Diastolic blood pressure (mmHg)	77.0 ± 8.1	76.1 ± 7.2	78.0 ± 9.1	0.40
METS (h/week) ^b^	38.8 (43.0)	35.0 (64.3)	39.2 (28.3)	0.97 ^d^
Depression				
Score	9.1 ± 5.7	7.8 ± 4.5	10.6 ± 6.7	0.09
Absent (%)	54.0	59.3	47.8	
Mild (%)	34.0	37.0	30.4	
Moderate (%)	10.0	3.7	17.4	
Severe (%)	2.0	0	4.4	0.27
Anxiety				
Score	23.3 ± 7.7	22.7 ± 6.6	24.1 ± 8.8	0.52
Mild (%)	24.0	22.2	26.1	
Mild to Moderate (%)	32.0	37.0	26.1	
Moderate to Severe (%)	44.0	40.7	47.8	0.71
Orthorexia				
Score	35.4 ± 5.4	35.9 ± 6.0	34.8 ± 4.7	0.51
Increased risk (%)	44.0	37.0	52.2	0.28
Eating disorders				
Score	4.5 (8.0)	3 (6.0)	6 (9.0)	0.014 ^d^
Increased risk (%)	8.0	0	17.4	0.024
Quality of life				
Total score	35.4 ± 6.0	37.5 ± 4.3	33.0 ± 6.8	0.007
Physical health score	16.0 ± 2.7	16.7 ± 2.1	15.3 ± 3.2	0.07
Mental health score	19.4 ± 3.9	20.8 ± 3.3	17.7 ± 4.0	0.004

NCGS, non-celiac gluten sensitive. ^a^ BMI = body mass index. ^b^ METS = metabolic equivalent of activity. ^c^ Data are expressed as mean ± SD or median (interquartile range). ^d^ Mann–Whitney test.

**Table 2:** Symptoms reported after the DBPC gluten challenge.

	Positive (NCGS), *n* = 27		Negative, *n* = 23	
	After Gluten	After Placebo	After Gluten	After Placebo
Total score, median (IQR)	17.0 (7.0)	2.0 (5.1)	5.4 (6.1)	14.0 (8.7)
Gastrointestinal symptoms, median (IQR)	14.9 (9.0)	2.0 (5.0)	4.0 (7.4)	10.7 (7.9)
Abdominal pain, *n* (%)	11 (40.1)	5 (18.5)	5 (21.7)	6 (26.1)
Bloating, *n* (%)	9 (33.3)	6 (22.2)	8 (34.8)	12 (52.2)
Meteorism, *n* (%)	7 (25.9)	7 (25.9)	5 (21.7)	10 (43.5)
Increased stool frequency, *n* (%)	7 (25.9)	2 (7.4)	3 (13.0)	2 (8.7)
Abdominal distention, *n* (%)	3 (11.1)	1 (3.7)	9 (39.1)	11 (47.8)
Extra-gastrointestinal symptoms, median (IQR)	0.0 (4.5)	0.0 (0.6)	1.0 (4.0)	2.7 (7.0)
Lesions of the oral cavity, *n* (%)	4 (14.8)	2 (7.4)	0 (0.0)	1 (4.3)
Dermatitis, *n* (%)	5 (18.5)	1 (3.7)	4 (17.4)	4 (17.4)
Headache, *n* (%)	4 (14.8)	2 (7.4)	4 (17.4)	5 (21.7)
Pain in the limbs, *n* (%)	0 (0.0)	1 (3.7)	1 (4.3)	3 (13.0)

IQR, interquartile range. Gastrointestinal symptoms were reported if the frequency was ≥20% in at least one of the four groups; extra-gastrointestinal symptoms were reported if the frequency was ≥10% in at least one of the four groups.

**Table 3:** Association between the positive response to the DBPC gluten challenge and different variables in a multiple logistic regression model.

	Crude	Adjusted ^a^
	OR; 95% CI, *p*	OR; 95% CI, *p*
EAT-26 score	0.87; 0.77–0.98, 0.022	0.87; 0.76–0.98, 0.021
Mental health score	1.28; 1.05–1.56, 0.011	1.30; 1.06–1.59, 0.009
Gut microbiota		
*Bacteroides*	0.94; 0.88–1.00, 0.048	0.94; 0.88–1.00, 0.050
*Blautia*	1.20; 1.04–1.38, 0.008	1.23; 1.05–1.44, 0.009
*Dorea*	0.93; 0.84–1.04, 0.18	0.93; 0.83–1.03, 0.17
*Parabacteroides*	0.35; 0.16–0.76, 0.007	0.34; 0.15–0.76, 0.007
*Actinomyces* ^b^	1.10; 0.94–1.27, 0.227	1.11; 0.95–1.31, 0.182
*Streptococcus* ^c^	Inf., *p* = 0.002	Inf., *p* = 0.004
Gut mycobiota		
*Debaryomyces* ^b^	0.61; 0.22–1.71, 0.34	0.58; 0.19–1.83, 0.36
*Kluyveromyces*	3.05; 0.49–19.0, 0.22	2.89; 0.48–17.5, 0.25
*Rhodotorula* ^b^	1.10; 0.96–1.27, 0.17	1.13; 0.96–1.34, 0.14

EAT-26, Eating Attitude Test-26. ^a^ Adjusted for age, gender, and education level. ^b^ Considering 0.01 of relative frequency as the unitary increase. ^c^ Since none among placebo-responsive individuals showed streptococci in their gut microbiota, the OR was = +∞; standard errors and *p*-values were computed by a resampling method (the jackknife technique).

## Data Availability

The data presented in this study are available on request from the corresponding author.

## Supplementary Materials

The following are available online at https://www.mdpi.com/article/10.3390/nu13041260/s1, Figure S1: Microbiota and mycobiota α-diversity by response to the DBPC gluten challenge test.
