# Supplementary material for: Non-Celiac Gluten/Wheat Sensitivity: Clinical Characteristics and Microbiota and Mycobiota Composition by Response to the Gluten Challenge Test
Source: Nutrients. 2021 Apr 12;13(4):1260. doi: 10.3390/nu13041260 (PMC8070191; doi:10.3390/nu13041260)

Figure Supplementary 1. Microbiota and mycobiota α-diversity by response to the DBPC gluten challenge test

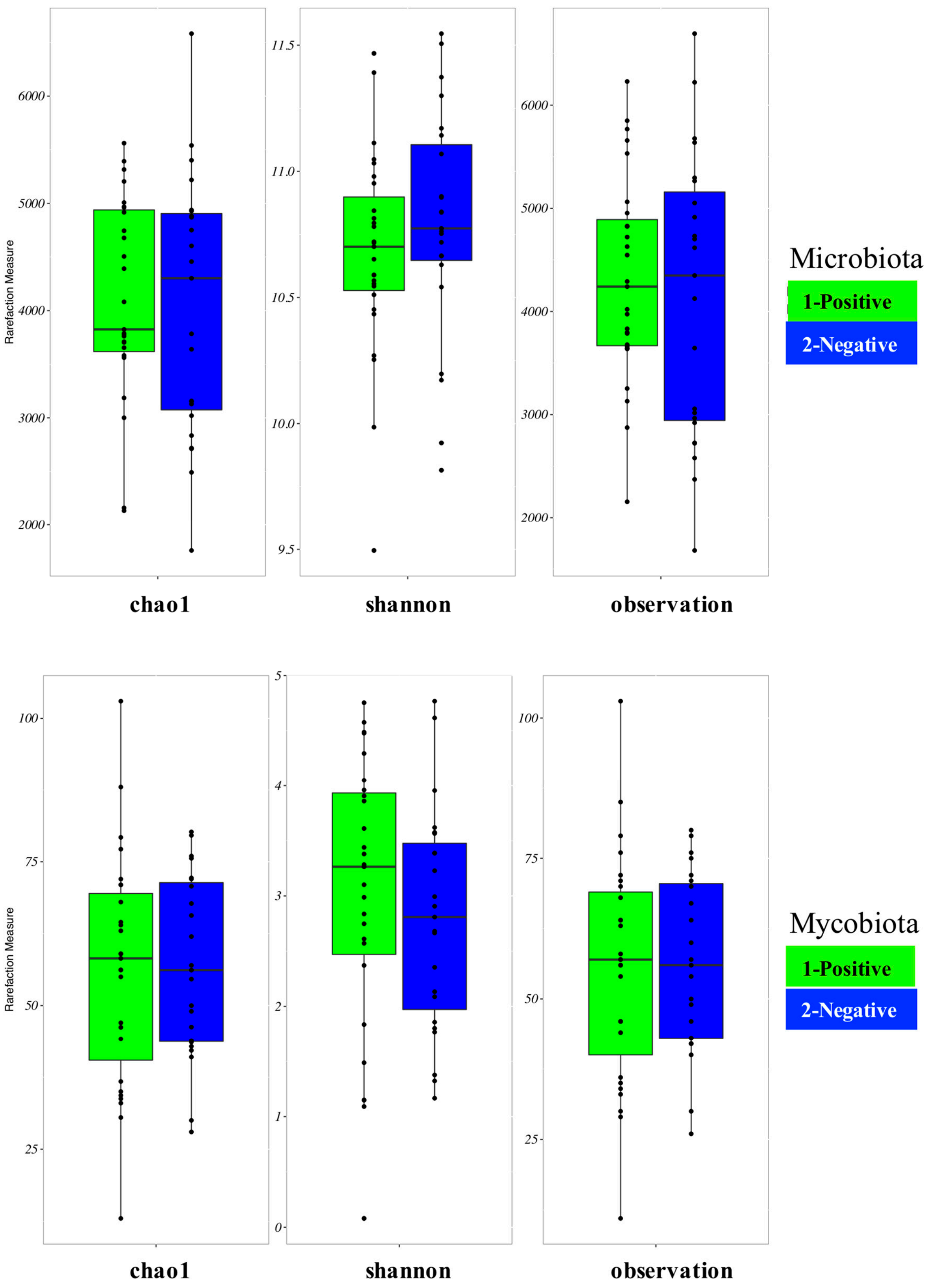

Supplement: Supplementary file 1 [file nutrients-13-01260-s001.pdf]
